# Feasibility and reliability of an automated controller of inspired oxygen concentration during mechanical ventilation

**DOI:** 10.1186/cc13734

**Published:** 2014-02-19

**Authors:** Kaouther Saihi, Jean-Christophe M Richard, Xavier Gonin, Thomas Krüger, Michel Dojat, Laurent Brochard

**Affiliations:** Intensive Care Unit, Department of Anesthesiology, Pharmacology and Intensive Care, Geneva University Hospital, Geneva, Switzerland; Dräger Medical GmbH, Lübeck, Germany; Grenoble Institut of Neurosciences (GIN)—INSERM U836 & Joseph Fourier University, Grenoble, France; Critical Care Department, St Michael’s Hospital, Toronto; InterDepartmental Division of Critical care Medicine University of Toronto, Toronto, Canada

## Abstract

**Introduction:**

Hypoxemia and high fractions of inspired oxygen (FiO_2_) are concerns in critically ill patients. An automated FiO_2_ controller based on continuous oxygen saturation (SpO_2_) measurement was tested. Two different SpO_2_-FiO_2_ feedback open loops, designed to react differently based on the level of hypoxemia, were compared. The results of the FiO_2_ controller were also compared with a historical control group.

**Methods:**

The system measures SpO_2_, compares with a target range (92% to 96%), and proposes in real time FiO_2_ settings to maintain SpO_2_ within target. In 20 patients under mechanical ventilation, two different FiO_2_-SpO_2_ open loops were applied by a dedicated research nurse during 3 hours, each in random order. The times spent in and outside the target SpO_2_ values were measured. The results of the automatic controller were then compared with a retrospective control group of 30 ICU patients. SpO_2_-FiO_2_ values of the control group were collected over three different periods of 6 hours.

**Results:**

Time in the target range was higher than 95% with the controller. When the 20 patients were separated according to the median PaO_2_/FiO_2_ (160(133-176) mm Hg versus 239(201-285)), the loop with the highest slope was slightly better (*P* = 0.047) for the more-hypoxemic patients. Hyperoxemia and hypoxemia durations were significantly shorter with the controller compared with usual care: SpO_2_ target range was reached 90% versus 24%, 27% and 32% (*P* < .001) with the controller, compared with three historical control-group periods.

**Conclusion:**

A specific FiO_2_ controller is able to maintain SpO_2_ reliably within a predefined target range. Two different feedback loops can be used, depending on the initial PaO_2_/FiO_2_; with both, the automatic controller showed excellent performance when compared with usual care.

**Electronic supplementary material:**

The online version of this article (doi:10.1186/cc13734) contains supplementary material, which is available to authorized users.

## Introduction

Oxygen is essential for life. As has any drug, it has consequences in case of under- and overdosing. In adult intensive care patients, hypoxemia is a primary preoccupation for all clinicians. The consequences of hyperoxemia are more often neglected because they have been poorly explored. Several clinical observations have suggested that liberal administration of oxygen can be toxic [1–3]. Hyperoxia induces the constitution of free oxygen radicals that may cause endothelial cell injury and increases the presence of inflammatory cells [4]. It can lead to absorption atelectasis in lung regions with low ventilation-to-perfusion ratios [5]. In adult intensive care patients, it has been shown that exposure to hyperoxemia may be harmful in specific populations. In post-cardiac arrest patients, arterial hyperoxemia was independently associated with in-hospital mortality, to an extent comparable to hypoxemia [6]. In nonventilated severe COPD patients with exacerbation, high FiO_2_ can be responsible for hypercapnia but also increased mortality [7]. In patients with severe traumatic brain injury, hyperoxemia is associated with increased mortality and worse outcomes [8].

Based on these concerns and the possibility that optimizing oxygenation targets may improve patients’ outcome, systems for automatic adjustment of FiO_2_ based on SpO_2_ measurement might be of great value to optimize care. With the use of pulse oximetry and computer technology, several attempts have been made to automate the adjustment of FiO_2_, especially in neonatology, because of the frequent and unpredictable change of oxygenation and risks of hyperoxemia in premature babies [9–12]. In adults, preliminary attempts at closed-loop control of oxygenation were developed and used in military trauma patients, as well as for titrating the FiO_2_ for COPD patients requiring long-term oxygen therapy [13–15]. These systems proved a reduction in oxygen use without inducing hypoxemia compared with conventional adjustments. Recently, an automated oxygen-flow titration was tested on healthy subjects during induced hypoxemia and showed a significant reduction of hypoxemia and hyperoxemia compared with classic constant-flow oxygen administration [16]. Last, a recent mode of ventilation allows full control of both pressure-targeted breaths and the level of FiO_2_ in a closed-loop manner. Two recent clinical studies showed the feasibility of this technique [17, 18].

To overcome the challenges of continuously maintaining an adequate oxygenation in adult ICU patients, we developed an automated oxygen-controller prototype that aims to maintain the measured SpO_2_ in a predefined target range [92% to 96%]. For this system, we defined two different FiO_2_-SpO_2_ feedback profiles with the hypothesis that the more-severely hypoxemic patients, because of intrapulmonary shunt, are less sensitive to FiO_2_ changes and need larger changes in FiO_2_ than do less-hypoxemic patients. The first aim of the current study was to test and compare these two different SpO_2_-FiO_2_ profiles in patients with different degrees of hypoxemia to maintain SpO_2_ in the predefined target range [92% to 96%]. To evaluate the clinical impact of the system, we also compared the results obtained with these two profiles of the FiO_2_ controller with usual care based on a comparable historical control group.

## Materials and methods

### Study design and patients

The study was conducted in the medical-surgical ICU of Geneva University Hospital. The first part of the study was a prospective trial performed in 20 ICU patients, and the second part included a retrospective analysis of consecutive admitted patients between September and October 2011 in the same ICU. The two parts of the study were accepted by the Ethics Committee of the hospital [The Ethic Committee and Research on Human Beings (CEREH), research project number 12089(NAC12040)].

For the first part, signed informed consents were obtained from the patient when possible or from the family, and from the attending physician. For both parts, inclusion criteria were similar and mechanically ventilated patients for more than 48 hours after ICU admission older than 18 years old. Patients with severe acidosis (pH ≤7.20), hemodynamic instability, serum lactate > 3mmol/L, or need for norepinephrine infusion ≥0.5 μg/kg/min, pregnant, or with intracranial hypertension were not included. Concerning the second part, SpO_2_ had to be recorded continuously to ensure the selection of the patient for the control group.

### The automated FiO_2_ controller

The FIO_2_-controller prototype tested in the present study included software implemented in a medical PC connected via RS-232 serial links to a ventilator (Evita XL; Dräger Medical, Lübeck, Germany) and to a pulse oximeter (Radical 7; Masimo Corp, Irvine, CA, USA) set at an averaging interval of 2 seconds. A probe was placed on the finger of the patient while we used an ear probe in case of poor perfusion, as indicated by the perfusion index of the Masimo. The perfusion index (PI) which is the ratio of the pulsatile blood flow to the nonpulsatile or static blood in peripheral tissue, was calculated continuously by the Masimo. A threshold of low signal and unreliable measurement was defined as signal index quality (SIQ) <0.30, where the latter represents Masimo’s quality indicator in case of extremely low perfusion and motion conditions [19]. When SIQ was <0.30, the FiO_2_ controller kept the last FiO_2_ before this low SIQ value.

The serial link connected to the pulse oximeter allowed a continuous recording of SpO_2_ values, as well as heart rate, perfusion index, SIQ, and SpO_2_ alarms every second. With the same frequency, FiO_2_ values measured on the inspiratory nozzle and set on the ventilator were acquired from the ventilator. The proposed FiO_2_ adjustments were indicated by an acoustic signal and displayed on the screen of the medical PC every 30 seconds. The purpose of the present study was to test the reliability of the system working in an open loop. To achieve this goal, a fully dedicated ICU research nurse executed the adjustments on the ventilator. He could deviate from any proposal if it was considered to be unsafe, according to his clinical judgment.

The target for the controller is the midpoint between the high (96%) and low (92%) SpO_2_ targets (that is, 94%). The automatic FiO_2_ controller compares the measured SpO_2_ with the target 94% and calculates the difference to control the delivered FiO_2_ set to the patient.

The delivered FiO_2_ depends on the selected version of the algorithm. These latter are two tables that define for each SpO_2_ deviations (ΔSpO_2_) an FiO_2_ step change (either increase or decrease) to be applied to the current FiO_2_. These two different tables define the two slopes of SpO_2_-FiO_2_ tested in this study. The difference is based on the fact that we hypothesized that, for severely hypoxemic patients, a larger change or step in FiO_2_ is required than for less-hypoxemic patients because intrapulmonary shunt makes those patients less “sensitive” to FiO_2_ changes.

After changing FiO_2_, a predefined time of 30 seconds has to expire before changing FiO_2_ is allowed. To react immediately in case of a severe hypoxemic event, the controller applies 100% of FiO_2_ when SpO_2_ <85%. This reaction is the same in the two versions of the algorithm.

For avoiding instabilities (that is, oscillations, overshoots), the reaction of the FiO_2_ controller is dampened based on physiological and technical delays. Because of this dampening of the controller, early adjustments every 30 seconds were possible. The controller is based on a conventional Proportional-Integral-Derivative (PID) control using both the SpO_2_-FiO_2_ slopes and ΔFiO_2_ step changes, based on an estimated effective FiO_2_.

### Study protocol

The study was composed of two parts: a prospective trial and a retrospective analysis.

#### First part, prospective trial

The first part of the study consisted of a prospective crossover trial that aimed to compare the usefulness of two feedback open-loop profiles for the FiO_2_ controller. The trial corresponded to two 3-hour periods applied in randomized order, with FiO_2_ adjusted according to each profile by a research nurse. As the main difference between the two profiles is the SpO_2_/FiO_2_ slope, we tested the clinical difference of using these two different slopes. During all study periods, the SpO_2_ target range was 92% to 96%. This range was consistent with previous clinical publications on automatic FiO_2_ controllers [13, 14, 16, 20]. It was considered a reasonable compromise that combines safety (limiting risk of hypoxemia) and efficacy to limit FiO_2_ in comparison to usual care, and which was also used in the control ICU. This was important for the comparison between the two groups in the study (study group and historical control group). A research nurse was fully dedicated for the FiO_2_ adjustments and remained at the bedside during each trial. Meanwhile, patients continued to receive usual care and ventilator parameters such as positive end-expiratory pressure (PEEP), were kept constant unless the clinician asked for changes. In two patients, a change of PEEP was required.

All patients were ventilated with the same ventilator (Evita XL) and were randomly allocated to an order for the two profiles by opening a sealed envelope. During the recordings, endotracheal suctioning could be needed. Before any suctioning, FiO_2_ was increased to 100%. This was obtained automatically in the first five patients (preoxygenation procedure function of the ventilator). However, this approach was not consistently used by the nurses because it was not a systematic standard approach for all patients. Therefore, we decided to recommend doing it manually in the 15 other patients, with FiO_2_ subsequently decreased by following the FiO_2_ controller suggestions. These episodes produced major changes in FiO_2_ and SpO_2_ (especially in the high range) over a short period, introducing noise in the signal and reducing the sensitivity of the comparison. We decided against keeping it in the comparison because we were expecting only small changes between the two profiles. We therefore removed for the comparison of the two profiles a period of 15 minutes for each episode of suctioning (it usually took between 5 and 10 minutes to come back to the preceding level) corresponding to the preoxygenation and suctioning maneuvers.

For each patient, we selected the blood gases and ventilation parameters measured at baseline (in the morning). The 20 patients were separated into two groups of 10 according to the median PaO_2_/FiO_2_ ratio: a moderately hypoxemic group with PF >188 mm Hg and a severely hypoxemic group with PF ≤188 mm Hg.

#### Second part

The second part of the study consisted of a retrospective data collection of an historical group composed of 30 patients admitted in the ICU before the start of the clinical protocol and ventilated at least 48 hours. The nurse:patient ratio during this period was 1:1 or 1:2, depending on the severity of the patient’s condition. Concerning FiO_2_ adjustments in the ICU, no explicit limitations were placed on the usual care, except a prescribed low SpO_2_ threshold for all patients. Thus, FiO_2_ settings in the historical control group were dependent on the physician or nurse in charge and could be reduced to 21% if necessary. Data were collected from a patient data-management system (Centricity Critical Care Clinisoft GE Healthcare) over three different periods of 6 hours (at admission, after 24 hours, at day 7). In this group, SpO_2_ values were recorded every 1 to 2 minutes. We were especially interested to the data obtained after 24 hours and at day 7, because our patients in the first part were studied after several days of mechanical ventilation. Patients’ identifying information was removed to keep them completely anonymous. In this control group, a subgroup of 17 patients had a minimal clinical threshold of SpO_2_ ≥92% specifically ordered by the clinician until the day 7 after admission; this subgroup was also compared with the FiO_2_ controller because the latter has the same low threshold for SpO_2_.

We could not precisely identify the suctioning periods in the control group, and therefore, for this analysis, suctioning maneuvers, and preoxygenation periods were kept in both groups (study group and the historical control group) for the analysis. For the control group, SpO_2_ was measured with a pulse-oximetry system (Intellivue MP70 monitor; Philips Medical Systems, Amsterdam, The Netherlands). SpO_2_ data recorded from the system contained very low values, which carried a high probability of not being real. This hypothesis was confirmed when compared with Masimo’s recordings, in which SpO_2_ values were almost always ≥80%. We therefore defined aberrant values as SpO_2_ <80% as corresponding to erroneous measurements or artifacts, and we removed them from all the recordings of this group.

### Patient data and analysis

For both groups, we collected the same baseline characteristics including the Acute Physiology and Chronic Health Evaluation (APACHE) II and the Simplified Acute Physiology Score (SAPS) II at the day of admission. Blood gases and care procedures were documented from nursing and medical records. The first arterial blood gases at the day of admission for the historical group and in the morning for the study group were selected to define the baseline values of pH, PaO_2_, PaCO_2_, SaO_2_, and to calculate the PaO_2_/FiO_2_ ratio. Ventilator settings and modes in addition to monitored measurements, including SpO_2_ and FiO_2_, were recorded.

Times with SpO_2_ above, within, and below the target range [92% to 96%] were reported as percentage of the recorded time. These latter defined, respectively, hyperoxemia (SpO_2_ ≥97%), normoxemia (SpO_2_ ≥92% and SpO_2_ ≤96%), and hypoxemia (SpO_2_ ≤91%). These percentages of time were used to compare the two profiles in the first part of the study and to compare the FiO_2_ controller and the control groups in the second part. The differences between the two slopes studied in the first part of the study were considered small enough to justify grouping together all data obtained with the FiO_2_ controller. The percentage of time spent within the target range was the primary outcome variable of efficacy, and the percentage of time spent outside the target was the outcome variable of safety.

### Statistical analysis

Statistical analysis was performed by using SPSS (SPSS 16.0; SPSS Inc, Chicago, IL, USA). Descriptive statistics (median and 25th and 75th percentiles) were used to summarize demographic characteristics and ventilation and blood gases baseline values. SpO_2_ percentages were presented as means with standard deviations. In the first part of the study, a Mann-Whitney *U* test was used to determine whether baseline characteristics (ventilation, blood gases, scores) were significantly different between the two groups of 10 patients separated on the median value of the PaO_2_/FiO_2_ ratio. We performed pairwise comparisons by using the Wilcoxon test to compare the two profiles in each group. In the second part, a *t* test was used to determine the significance of the difference between the study group and the historical group.

## Results

### First part

#### Patients

Twenty-two patients were enrolled in the study (sixteen men and six women), and two patients could not complete the study (the first one experienced self-extubation after 2 hours of recordings, and the second one’s condition was severely worsened before starting the trial). All 20 patients tolerated the adjustments and completed both tests. We classified the 20 included patients into two categories of hypoxemia, according to their median PaO_2_/FiO_2_ ratio. Table 1 describes the characteristics of the two groups. They were comparable except for an older age in the moderately hypoxemic patients. Table 2 shows ventilation parameters and arterial blood gases. Tidal volume was higher and FiO_2_ lower in the moderately hypoxemic patients. For arterial blood gases, only oxygenation was significantly different between the two groups (*P* < 0.001).

**Table 1:** **Baseline characteristics of the patients**

Variable	Severe hypoxemia	Moderate hypoxemia	Ρ value
	(***n*** = 10)	(***n*** = 10)	
Age, years	65 (49-69)	76 (72-83)	0.029
Sex, male/female	7/3	7/3	
Height, cm	175 (171-183)	170 (161-177)	0.190
Weight, kg	71 (66-77)	66 (58-75)	0.315
Heart rate, beats per minute	88 (74-105)	84 (75-96)	0.393
APACHE II, at ICU admission	27 (24-30)	31 (26-33)	0.165
SAPS II, at ICU admission	53 (51-58)	56 (43-66)	0.684
SOFA, at day of recording	7 (7-9)	11 (9-13)	0.052
RASS, at day of recording	-4 (-4 to -1)	-1 (-3 to -1)	0.631
Mechanical ventilation, days	4 (2-7)	3 (1-5)	0.393
ICU stay, days	4 (2-7)	4 (1-8)	0.579
**Respiratory diagnosis,** ***n*** **(%)**			
Pneumonia	3 (23)	1(10)	
Acute pulmonary edema	1 (8)	0 (0)	
COPD	4 (31)	2 (20)	
ARDS	3 (23)	0 (0)	
Other	2 (15)	7 (70)	
**Equipment,** ***n***			
Endotracheal tube/tracheostomy	9/1	9/1	
Diameter of the tube, mm	8.0 (7.5-8.0)	7.5 (7.5-8.0)	0.165

APACHE, Acute Physiology and Chronic Health Evaluation; ICU, intensive care unit; SAPS, Simplified Acute Physiology Score; RASS, Richmond Agitation Sedation Scale. ARDS, acute respiratory distress syndrome; COPD, chronic obstructive pulmonary disease. “Other” patients had cardiogenic shock (three), cardiac surgery, spine surgery, cardiorespiratory arrest, or liver failure. One patient could be considered to have a normal lung.

**Table 2:** **Ventilation and arterial blood gases of the patients**

Variable	Severe hypoxemia	Moderate hypoxemia	Ρ value
	(***n*** = 10)	(***n*** = 10)	
**Ventilator mode at study inclusion, n (%)**			
Pressure support ventilation	14 (70)	14 (70)	
Pressure control ventilation	3 (15)	5 (25)	
Volume assist control ventilation	2 (10)	1 (5)	
Synchronized Intermittent Mandatory Ventilation	1 (5)	0 (0)	
**Ventilator settings (in the morning)**			
MV exp,vL	10 (10-12)	8 (7-10)	0.123
Tidal volume, ml/kg predicted body weight	7 (6-8)	9 (7-10)	0.011
Positive end-expiratory pressure, cm H_2_O	5 (5-7)	7 (5-8)	0.393
Peak inspiratory pressure, cm H_2_O	20 (17-25)	22 (18-25)	0.853
Inspired fraction of oxygen, %	40 (40-45)	30 (25-35)	0.001
**Arterial blood gases (in the morning)**			
pH	7.45 (7.39-7.48)	7.40 (7.38-7.44)	0.247
PaO_2_, mm Hg	69 (63-71)	76 (72-79)	0.005
PaCO_2_, mm Hg	38 (35-54)	39 (35-42)	0.796
SaO_2_, %	94 (93-95)	97 (96-97)	0.001
PaO_2_/FiO_2_, mm Hg	160 (133-176)	239 (201-285)	<0.001

#### Hypoxemia, normoxemia, and hyperoxemia

**Figure 1:**
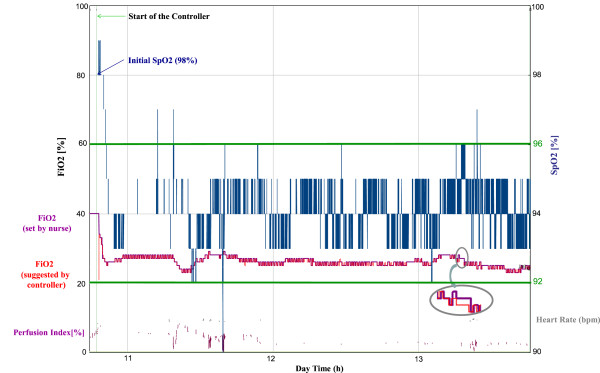
**Example of a patient’s recording over a 3-hour period, displaying five signals respectively described from top to bottom: SpO**_**2**_ **levels over time (blue line, bold); FiO**_**2**_ **steps set by the nurse (purple) and proposed by the FiO**_**2**_ **controller (red); at the bottom, heart rate (grey) and perfusion index (purple) from Masimo.** Please note that the grey circles indicate an example in which FiO_2_ set and FiO_2_ proposed were slightly divergent: this indicated a situation in which the nurse did not fully follow the FiO_2_-controller’s suggestions.

Figure 1 shows an example of a patient’s recording: normoxemia was maintained by the FiO_2_ controller during 98.0% of the recording time; hyperoxemia represented 2.0%, and hypoxemia, 0.1%. FiO_2_ set by the research nurse and suggested by the FiO_2_ controller were continuously recorded. More than 98% of the time, the research nurse followed the FiO_2_-controller suggestions.

Table 3 compares the amount of time that patients spent within and outside the target SpO_2_. Periods corresponding to an absence of signal and when it was not valid were also recorded. According to these criteria, we compared the two profiles (slopes of response designed for severely hypoxemic and moderately hypoxemic patients) in each group.

**Table 3:** **Percentage of time spent in different SpO**_2_ **ranges in each group according to the two controller profiles**

Group	Severe hypoxemia group			Moderate hypoxemia group		
	(***n*** = 10)			(***n*** = 10)		
	SH-profile	MH-profile	***P*** value	SH-profile	MH-profile	***P*** value
**Time with no signal (%)**	0.1 ± 0.2	0.1 ± 0.2	0.686	0.4 ± 0.5	0.1 ± 0.1	0.050
**Time with SIQ ≤ 0.3 (%)**	0.1 ± 0.2	0.1 ± 0.2	1.000	0.3 ± 0.6	0.0 ± 0.0	0.072
**Time with hypoxemia (SpO**_**2**_ **≤ 91%) (%)**	1.7 ± 2.2	1.9 ± 1.9	0.859	1.2 ± 1.0	1.0 ± 1.0	0.721
**Time with normoxemia (SpO**_**2**_ **(92% to 96%)) (%)**	96.7 ± 4.2	95.2 ± 4.8	0.047	95.1 ± 4.2	97.3 ± 2.8	0.074
**Time with hyperoxemia (SpO**_**2**_ **≥ 97%) (%)**	1.4 ± 2.1	2.8 ± 3.0	0.059	3.0 ± 3.0	1.6 ± 2.1	0.074

SIQ, Signal Index Quality; SH, severely hypoxemic; MH, moderately hypoxemic.

The percentage of time spent in the target range was higher than 95% in all cases. The severely hypoxemic profile was slightly better (*P* < 0.05) for the more-hypoxemic patients (PaO_2_/FiO_2_ < 188) to keep them in normoxemia. The number of suctioning episodes were calculated in each group and reported in Additional file 1: Table S1. For these maneuvers, no specific protocol was defined.

### Second part

#### Patients

Thirty patients were included in the analysis for the control group. All patients were mechanically ventilated within the first 24 hours after ICU admission. Data about severity of illness, respiratory diagnosis, and demographic characteristics are given in Additional file 2: Table S2. A subgroup of 17 patients had a prescribed lower SpO_2_ threshold of 92% (that is, identical lower SpO_2_ threshold than with the FiO_2_ controller), and was also analyzed separately from the historical group. Additional file 3: Table S3 presents ventilation and blood gases for both groups.

#### Group comparison

**Figure 2:**
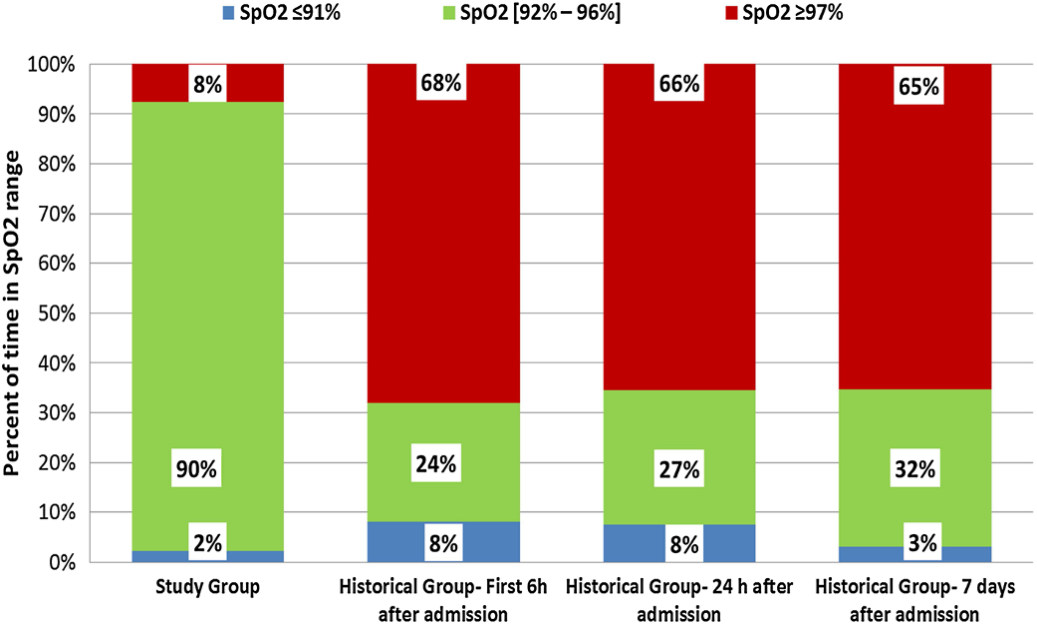
**Percentages of time within the predefined SpO**_**2**_ **ranges during three periods (first 6 hours after admission, after 24 hours, and after 7 days) in the historical group compared with the study group.**

**Figure 3:**
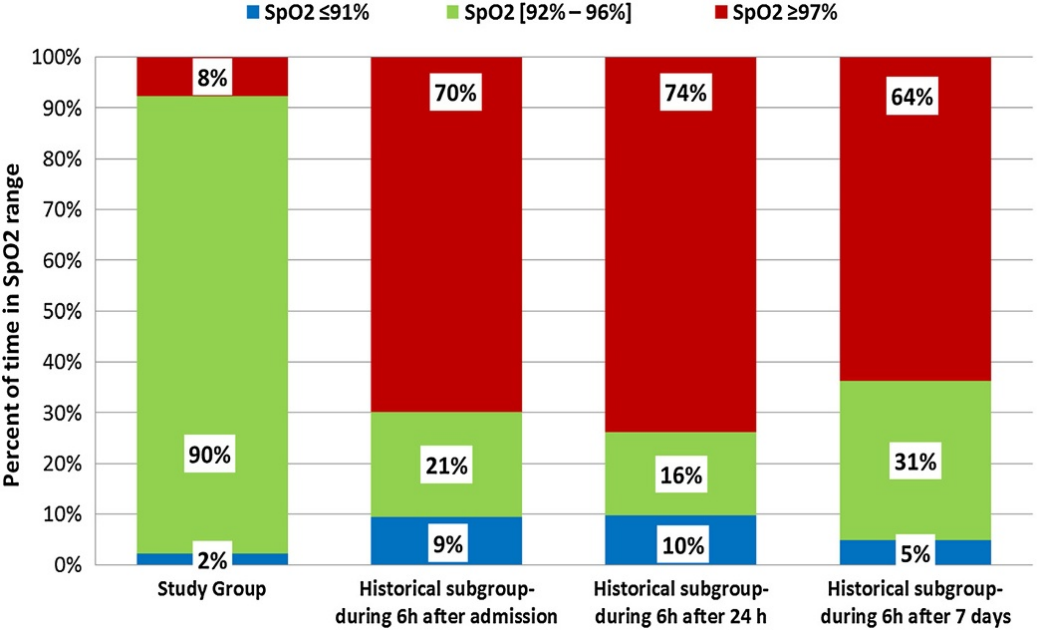
**Percentages of time within the predefined SpO**_**2**_ **ranges during three periods (first 6 hours after admission, after 24 hours, and after 7 days) in the historical subgroup with a lower SpO**_**2**_ **threshold at 92% compared with the study group.**

We compared the study group with the historical groups to assess the efficiency of the FiO_2_ controller in maintaining the SpO_2_ within the target range and reducing time in hyperoxemia and hypoxemia. For both historical groups, we selected three periods of 6 hours: after admission in the ICU, after 24 hours, and after 7 days (*n* = 25), and the results are presented in Figures 2 and 3, which illustrate the distribution of the time spent in the different SpO_2_ ranges in the FiO_2_ controller group and in the control groups. All comparisons between the study group and both historical groups were significant, showing a shorter time spent both in hyperoxemia and in hypoxemia with the automatic FiO_2_ controller (see Additional file 4: Table S4). In the historical group, a slight decrease in hypoxemia (SpO_2_ ≤91%) and hyperoxemia (SpO_2_ ≥97%) periods was found after 7 days after admission compared with the other two periods.

## Discussion

The study showed that a specific open-loop FiO_2_ controller is able to maintain SpO_2_ reliably within a predefined target range. The time spent in the defined range is much higher than in clinical practice, because of reduced time both in hyperoxemia and in hypoxemia. Although differences between the two FiO_2_-SpO_2_ slopes of responses are relatively small, each profile was well adapted to each category of patients.

The historical group in our study, in accordance with the literature, suggests that the situation of oxygenation control can be improved. In ICUs, considerable variations exist in the attitude toward oxygen management. A survey among New Zealand and Australian intensivists showed a large variation in practices [21]: for instance, for a ventilated acute respiratory distress syndrome patient, 37% of respondents would not allow SaO_2_ of <85% for ≤15 minutes, and 28% would not allow SaO_2_ <90% for >24 hours. Hypoxemia is a major concern to clinicians, whereas hyperoxemia is often left out of consideration. A Canadian questionnaire study showed that most respondents prevent much more hypoxemia than hyperoxemia [8]. A recent Dutch study [22] showed that, in terms of minimizing hyperoxemia, intensivists are simply guided by reducing FiO_2_ to levels presumed to be nontoxic, with little concern for PaO_2_ level.

Several recent clinical observations have, however, suggested that liberal administration of oxygen can be toxic. In an observational multicenter study concerning patients admitted after resuscitation from cardiac arrest, those exposed to hyperoxemia (PaO_2_ ≥300 mm Hg) experienced increased mortality compared with both normoxemic and hypoxemic groups (PaO_2_ <60 mm Hg) [2]. Administering supplemental oxygen to coronary heart disease (CHD) patients with the goal of maintaining 100% saturation might result in vasoconstriction in the coronary circulation and hemodynamic instability [3]. In a general ICU population, both low PaO_2_ and high PaO_2_ during the first 24 hours after ICU admission were associated with hospital mortality, forming a U-shaped curve [1].

Another study found only an association between hypoxemia and increased in-hospital mortality [23]. Recently, Rachmale et al. [24] evaluated prospectively the electronic medical record screening of 289 ICU patients with acute lung injury to assess excessive oxygen exposure and its effect on pulmonary outcomes. Excessive FiO_2_ was defined as FiO_2_ >0.5, despite SpO_2_ >92%, and results showed that 74% of the included patients were exposed to it. The authors demonstrated a correlation between prolonged FiO_2_ exposure and worsening of oxygenation index at 48 hours and an association with longer duration of mechanical ventilation and ICU stay.

In our study, the potential of the automatic controller is best shown with the comparison of the time spent in hypoxemia with the historical subgroup. This subgroup had a prescribed lower SpO_2_ threshold of 92%, but no upper limitation. The FiO_2_ controller showed better results in preventing hypoxemia, at the same time, keeping the time with hyperoxemia to a minimum and thus maximizing time in normoxemia.

In accordance with current ICU practice, the monitoring of oxygenation was based on pulse oximetry that continuously and noninvasively measures SpO_2_. Pulse oximetry has been shown to be a reliable technique for measuring the oxygen level and reduces the frequency of blood gas analysis [25–28]. Pulse oximetry has limitations, as, for example, artifacts due to patient motion, and low perfusion [29, 30]. Among pulse-oximetry technologies, Signal Extraction Technology, as used by Masimo, seems to have superior performance compared with other pulse-oximetry technologies in terms of motion and artifact, false alarms, and data dropout [31–34]. In neonatology, such technology has been shown helpful in reducing severe retinopathy of prematurity in preterm infants treated with supplemental oxygen [35].

With the use of pulse oximetry and computer technology, several attempts have been made to automatize the adjustment of FiO_2_, especially in neonatology because of the frequent and unpredictable change of oxygenation and risks of hyperoxemia in premature babies [9–12, 19, 36]. This automation has rarely been proposed to adults to guide the clinician to the most appropriate FiO_2_, apart from research. A closed-loop control of oxygenation used in military trauma patients demonstrated its efficiency at reducing oxygen needs and showed that even severely injured trauma patients can be managed with FiO_2_ <0.30 [37]. Rees *et al*. [38] created a decision support system that provides advice about FiO_2_ setting, tidal volume, and frequency rate based on physiological models. The system has been tested retrospectively and prospectively in a few patients to evaluate its ability to provide appropriate FiO_2_ suggestions and has shown better FiO_2_ selection in comparison with attending clinicians in intensive care patients [39, 40]. A system that automatically controls oxygen administration during nasal oxygen therapy has been proposed, based on SpO_2_ measurements [16]. A fully controlled ventilation system was also compared with usual care in a randomized controlled trial of postoperative patients after cardiac surgery [18]. Both ventilation and FiO_2_ were automatically controlled with a target for SpO_2_ of 94% to 98%. The patients in the automated ventilation arm spent less time in nonacceptable ventilation zones, but very few details were specifically given concerning oxygenation.

We adapted an open-loop inspired oxygen control system for use in adults that has been recently tested successfully on intensive care neonates used in a closed-loop manner [41]. We designed two profiles, with the hypothesis that the more-hypoxemic patients, as defined by the lowest PaO_2_/FiO_2_ ratio, would be less sensitive to FiO_2_ changes because of intrapulmonary shunt. This was confirmed in the clinical study, although the differences between the two slopes were modest. A clinician could use the classic threshold of 200 mm Hg of PaO_2_/FiO_2_ ratio to select the best slope, but if the other slope were to be selected, the results would remain safe.

### Limits of the study

First, the automated FiO_2_-controller prototype tested in the present study presents some technologic limits because it depends on the reliability and the accuracy of SpO_2_ and adjusts only the FiO_2_. It does not adjust the PEEP level, for instance. Such a system must also contain alarms alerting the clinician when consistent and substantial changes in FiO_2_ are observed; otherwise, a risk would be to reduce the attentiveness of the caregiver and delay recognition of changes in respiratory function. These alarms were not specifically tested with the open loop. We excluded patients with hemodynamic instability to limit the risk of deterioration of the patient and did not face any problem because of low signal-quality measurement. The controller algorithm is also able to validate the SpO_2_ signal quality and enters into a fall-back state, keeping FiO_2_ constant. This condition must be investigated to test the reliability of the system in extreme conditions. Last, the experimental design gave us the unique opportunity to compare the system with usual care but could not permit us to assess the workload reduction expected with the automatic system. The 6-hour period tested in the present study is relatively short. When we investigated different time windows in the control group, however, they all looked very similar, suggesting that these 6-hour periods are meaningful and representative.

## Conclusion

The tested open-loop system allowed maintaining SpO_2_ within a target range and decreased hyperoxemia and hypoxemia periods in comparison with usual care. It could provide physiological and clinical benefits to patients. As with every automated system, it requires an understanding of its operation and vigilance. This study opens the perspective for a test in a closed loop in comparison with usual care.

## Key messages

- An automated FiO_2_ controller based on oxygen-saturation measurement is able to maintain SpO_2_ reliably in a safety-predefined range during mechanical ventilation of adult critically ill patients.
- The Automatic FiO_2_ controller exhibits excellent performance in adjusting FiO_2_ at different levels of baseline PaO_2_/FiO_2_ ratio.
- Automatic adjustment of FiO_2_ was able to maintain SpO_2_ in a predefined target range much better compared with a historical group of mechanically ventilated patients.

## Electronic supplementary material

Additional file 1: Table S1: Percentage of the recording time spent in the different ranges of SpO_2_ according to groups. (PDF 57 KB)

Additional file 2: Table S2: Numbers of suctioning in each group according to the two controller profiles. (DOC 30 KB)

Additional file 3: Table S3: Baseline characteristics of the historical groups. (DOCX 16 KB)

Additional file 4: Table S4: Ventilation and arterial blood gases of the historical groups. (DOCX 18 KB)

## Abbreviations

APACHE II: Acute Physiology and Chronic Health Evaluation II
ARDS: acute respiratory distress syndrome
COPD: chronic obstructive pulmonary disease
FiO2: fraction of inspired oxygen
ICU: intensive care unit
MH: moderately hypoxemic
PaCO2: carbon dioxide partial pressure
PaO2: arterial oxygen partial pressure
PaO2/FiO2 ratio: ratio of arterial oxygen partial pressure to fraction of inspired oxygen
PEEP: positive end-expiratory pressure
RASS: Richmond Agitation Sedation Scale
SaO2: arterial oxygen saturation
SAPS II: Simplified Acute Physiology Score II
SH: severely hypoxemic
Signal IQ: Signal Index Quality
SpO2: arterial oxygen saturation by pulse oximetry.
