# Supplementary material for: Feasibility and reliability of an automated controller of inspired oxygen concentration during mechanical ventilation
Source: Crit Care. 2014 Feb 19;18(1):R35. doi: 10.1186/cc13734 (PMC4031979; doi:10.1186/cc13734)
Supplement: Supplementary file 1 — Additional file 1: Table S1: Percentage of the recording time spent in the different ranges of SpO2 according to groups. (PDF 57 KB) [file 13054_2013_2861_MOESM1_ESM.pdf]

S1: Percentage of the recording time spent in the different ranges of SpO2 according to groups

Proportion of Time, %	Study group (n= 20)	Historical group (n= 30)						Historical subgroup with lower SpO2 threshold (n= 17)					
	6h of recording	First 6 h after admission*	P	At 24 h**	P	At Day 7*** (n=25)	P	First 6 h after admission *	P	At 24 h**	P	At Day 7***(n=14)	P
SpO2 of ≤91%, mean ±SD	2.3 ± 1.6	8.0 ± 13.6	0.002	7.6 ± 18.9	0.010	3.1± 9.4	0.072	9.4 ± 16.4	0.002	9.7 ± 24.0	0.003	5.0± 12.3	0.007
SpO2of [92% – 96%], mean ±SD	90.2 ± 4.9	23.9 ± 27.8	<0.001	26.9 ± 32.2	<0.001	31.7 ± 29.9	<0.001	20.8 ± 26.3	<0.001	16.5 ± 25.2	0.003	31.2 ± 27.3	<0.001
SpO2 of ≥97%, mean ±SD	7.7 ± 3.7	68.1 ± 32.5	<0.001	65.5 ± 35.8	<0.001	65.2 ± 32.3	<0.001	69.8 ± 31.8	<0.001	73.9 ± 33.9	<0.001	63.8 ± 31.7	<0.001
