# Supplementary material for: Feasibility and reliability of an automated controller of inspired oxygen concentration during mechanical ventilation
Source: Crit Care. 2014 Feb 19;18(1):R35. doi: 10.1186/cc13734 (PMC4031979; doi:10.1186/cc13734)
Supplement: Supplementary file 2 — Additional file 2: Table S2: Numbers of suctioning in each group according to the two controller profiles. (DOC 30 KB) [file 13054_2013_2861_MOESM2_ESM.doc]

Table S2: Number of suctioning in each group according to the two controller profiles

| Patient Group | Severe Hypoxemia Patients  (n=10) | | Moderate Hypoxemia Patients  (n=10) | |
| --- | --- | --- | --- | --- |
|  | SH-Profile | MH-Profile | SH-Profile | MH-Profile |
| Total | 12 | 11 | 5 | 7 |
