# Supplementary material for: Feasibility and reliability of an automated controller of inspired oxygen concentration during mechanical ventilation
Source: Crit Care. 2014 Feb 19;18(1):R35. doi: 10.1186/cc13734 (PMC4031979; doi:10.1186/cc13734)
Supplement: Supplementary file 3 — Additional file 3: Table S3: Baseline characteristics of the historical groups. (DOCX 16 KB) [file 13054_2013_2861_MOESM3_ESM.docx]

Table S3: Baseline characteristics of the historical groups

| Variable | Historical Group  (n=30) | Subgroup with SpO_2_ ≥92% (n=17) |
| --- | --- | --- |
| Age, yr | 58 (46-69) | 55 (47-69) |
| Sex , male/female | 20 / 10 | 10 / 07 |
| Height, cm | 171 (165-180) | 170 (165-172) |
| Weight, kg | 79 (61-87) | 70 (60-83) |
| Heart Rate, bpm | 82 (73-96) | 81 (72-94) |
| APACHE II, ICU admission | 26(21-32) | 28(22-33) |
| SAPS II , ICU admission | 51 (40-60) | 57 (45-65) |
| Mechanical Ventilation, days | 9 (6-14) | 7 (6-10) |
| ICU stay, days | 12(8-21) | 11(8-17) |
| **Respiratory Diagnosis, number (%)** |  |  |
| Pneumonia | 13 (39) | 9 (50) |
| Acute Pulmonary Edema | 2 (6) | 2 (11) |
| COPD | 5 (15) | 3 (17) |
| ARDS | 4 (12) | 1 (6) |
| Other | 9 (27) | 3 (17) |
| **Equipment, number** |  |  |
| Endotracheal Tube/ Tracheotomy | 30 / 0 | 17 / 0 |
| Diameter of the tube, mm | 7.5 (7.5-8.0) | 7.5 (7.0-7.5) |

Definition of abbreviations: APACHE, Acute Physiology and Chronic Health Evaluation; ICU, Intensive Care Unit; SAPS, Simplified Acute Physiology Score; RASS, [Richmond Agitation Sedation Scale; COPD, Chronic Obstructive Pulmonary Disease; ARDS, Acute Respiratory Distress Syndrome.](http://acronyms.thefreedictionary.com/Richmond+Agitation+Sedation+Scale)
