# Supplementary material for: Feasibility and reliability of an automated controller of inspired oxygen concentration during mechanical ventilation
Source: Crit Care. 2014 Feb 19;18(1):R35. doi: 10.1186/cc13734 (PMC4031979; doi:10.1186/cc13734)
Supplement: Supplementary file 4 — Additional file 4: Table S4: Ventilation and arterial blood gases of the historical groups. (DOCX 18 KB) [file 13054_2013_2861_MOESM4_ESM.docx]

Table S4: Ventilation and arterial blood gases of the historical groups

| Variable | Historical Group  (n=30) | Subgroup with SpO_2_ ≥92% (n=17) |
| --- | --- | --- |
| **Ventilator mode at study inclusion, number (%)** |  |  |
| Pressure Support Ventilation | 12 (40) | 6 (35) |
| Pressure Control Ventilation | 3 (10) | 3 (18) |
| Volume Assist Control Ventilation | 3 (10) | 3 (18) |
| Synchronized Intermittent Mandatory Ventilation | 12 (40) | 5 (29) |
| **Ventilator settings (in the morning)** |  |  |
| MV exp ,L | 9 (8-12) | 9 (8-11) |
| Tidal Volume, ml/kg predicted body weight | 8 (6-10) | 8 (6-9) |
| Positive End-Expiratory Pressure, cm H_2_O | 5 (5-6) | 5 (5-7) |
| Peak Inspiratory Pressure, cm H_2_O | 22 (19-33) | 20( 18-23) |
| Inspired Fraction of Oxygen | 60 (50-60) | 60 (50-60) |
| **Arterial Blood Gases (in the morning)** |  |  |
| pH | 7.30 (7.26-7.39) | 7.32 (7.27-7.39) |
| PaO_2_, mmHg | 108 (73-247) | 230 (71-334) |
| PaCO_2_, mmHg | 38 (34-45) | 37 (32-46) |
| SaO_2_, % | 98 (95-99) | 99 (94-99) |
| PaO_2_/FiO_2_, mmHg | 234 (150-446) | 344 (144-502) |
